# Upadacitinib in Crohn’s Disease: A Comprehensive Systematic Review of Efficacy and Safety

**DOI:** 10.7759/cureus.50657

**Published:** 2023-12-17

**Authors:** Aishwarya M Wodeyar, Nirav Pansuriya, Shahzeb Saeed, Alisha Lakhani, Sahil Sartaj, Naga Sathya Joshitha Keerthi, Akshara Guntur Bhuvika Raji, Bhavatharini S, Vaishali Wahane, Yeshika Thapa, Feven Abriha

**Affiliations:** 1 Clinical Pharmacology and Therapeutics, KS Hegde Medical Academy, Mangaluru, IND; 2 Medicine, Surat Municipal Institute of Medical Education and Research, Surat, IND; 3 Medicine, Army Medical College, Rawalpindi, PAK; 4 Medicine, Research MD, Vadodara, IND; 5 Medicine, Shantabaa Medical College, Amreli, IND; 6 Medicine, Melmaruvathur Adhiparasakthi Institute of Medical Sciences and Research, Melmaruvathur, IND; 7 Medicine, Danylo Halytsky Lviv National Medical University, Lviv, UKR; 8 Medicine, Bhaskar Medical College, Hyderabad, IND; 9 Medicine, Government Theni Medical College, Theni, IND; 10 Radiology, Ultrascan Diagnostic Center, Mumbai, IND; 11 Internal Medicine, Chitwan Medical College, Tribhuvan University, Bharatpur, NPL; 12 Medicine, Jimma University Medical School, Jimma, ETH

**Keywords:** gastroenterology and hepatology, safety, efficacy, systematic review, upadacitinib, crohn’s disease

## Abstract

Crohn’s disease (CD) presents a formidable challenge as a chronic inflammatory condition. This systematic review aimed to comprehensively assess upadacitinib, a novel Janus kinase (JAK) inhibitor, regarding its efficacy, safety, and mechanistic insights in CD treatment.

A thorough search of electronic databases identified studies investigating upadacitinib's impact on CD patients. Study characteristics, efficacy outcomes (clinical remission and endoscopic response), safety profiles, and mechanistic insights were extracted and qualitatively synthesized.

Methodological quality was assessed using established tools. The synthesis of three studies consistently demonstrated improvements in clinical remission rates and endoscopic outcomes in upadacitinib-treated patients. Adverse events, such as herpes zoster, intestinal perforation, non-melanoma skin cancer, adjudicated cardiovascular events, and anemia, were reported, necessitating vigilant safety monitoring.

Upadacitinib emerges as a promising therapeutic option for CD, supported by its observed clinical benefits and mechanistic implications. However, safety concerns underscore the importance of careful patient selection. These findings contribute to the ongoing discussion surrounding personalized treatment approaches for CD, emphasizing the need for further research to confirm its enduring efficacy and safety.

## Introduction and background

Crohn's disease (CD) is a chronic inflammatory bowel disease (IBD) that primarily affects the gastrointestinal tract. It is characterized by inflammation of the digestive or gastrointestinal (GI) tract, leading to a range of symptoms and complications. The exact cause of Crohn’s disease is unknown; however, several factors have been implicated in its development. These include a dysregulated immune system, altered microbiota, genetic susceptibility, and environmental factors [1-3]. It affects the gastrointestinal system transmurally from the mouth to the anus. In most cases, patients with this condition present with abdominal pain, fever, and clinical signs of bowel obstruction, such as mucus passage [4].

Despite advancements in therapeutic options, a subset of patients with CD experience inadequate responses to conventional treatments, leading to a pressing need for novel strategies that can effectively address the intricate interplay between inflammation, immune response dysregulation, and tissue damage inherent in the disease [5,6].

Upadacitinib, a novel Janus kinase (JAK) inhibitor, has emerged as a promising therapeutic candidate for the management of inflammatory disorders including Crohn’s disease. The JAK family of kinases plays a pivotal role in mediating signalling pathways downstream of various cytokine receptors implicated in immune response modulation [7,8].

The potential therapeutic utility of upadacitinib in the treatment of Crohn's disease stems from its ability to modulate key pathways involved in its pathogenesis. Specifically, by selectively inhibiting JAKs, upadacitinib has the capacity to decrease the production of pro-inflammatory cytokines, such as interleukins (IL-6, IL-12, IL-23, and interferons {IFN-γ}), which are crucial to the maintenance of chronic inflammation in CD. This targeted approach holds promise for achieving remission and improving patient quality of life, particularly for those who do not respond optimally to existing treatment modalities [9].

Clinical trials have demonstrated that patients with moderate-to-severe Crohn's disease responded favorably to upadacitinib induction and maintenance medication compared to a placebo, and the benefit-to-risk ratio was deemed favorable [10-12].

The objective of this systematic review was to evaluate the efficacy, safety, and potential mechanisms of upadacitinib in the treatment of Crohn's disease. Through a comprehensive analysis of the available evidence, we aimed to provide a comprehensive understanding of the potential of upadacitinib as an innovative therapeutic strategy, shedding light on its ability to modulate inflammation, restore immune homeostasis, and alter the natural course of the disease [13].

In the subsequent sections, we delve into the methodology employed to identify and select relevant studies, present the findings from the selected studies, discuss their implications, and outline the limitations of the systematic review process. By systematically examining upadacitinib’s potential as a therapeutic intervention in Crohn’s disease, we aimed to contribute to the ongoing discourse on tailored treatment approaches for CD patients, offering insights that may guide clinical decision-making and stimulate further research in this evolving field.

## Review

Subjects and methods

Research Question and Objectives

The primary aim of this systematic review is to comprehensively assess the efficacy and safety of Upadacitinib in the treatment of Crohn's disease. The specific objectives included evaluating the available evidence on the effects of Upadacitinib on clinical outcomes and adverse events.

Search strategy: To identify relevant studies, a comprehensive search strategy was devised and implemented. Electronic databases such as PubMed, Embase, and the Cochrane Library were systematically searched using a combination of keywords and Medical Subject Headings (MeSH) terms related to “upadacitinib” and “Crohn’s disease.” The studies from 2013 to 2023 were included in the study. In addition, gray literature sources, conference proceedings, and reference lists of the included studies were scanned to identify any additional relevant studies that might not have been captured by the primary search.

Inclusion and Exclusion Criteria

Studies were included based on predefined inclusion criteria. Observational studies and clinical trials from 2013 to 2023 investigating the effects of upadacitinib in patients with Crohn’s disease and in the English language were included. Also, studies reporting the efficacy and safety of upadacitinib in Crohn’s were considered. Studies that did not report relevant outcomes, non-human studies, or those not published in English were excluded. Abstracts, books, and documents were also excluded from the study.

A total of 155 articles were obtained through the literature search, of which, 52 were removed due to duplication. Title and abstract screening identified 103 articles for full-text examination, which subsequently led to the inclusion of three publications. Articles that were relevant to the research topic were included and the rest were filtered out on the basis of exclusion criteria. Articles not pertaining to the aim of the study were not included. Therefore, the evidence base comprised three publications, pertaining to two randomized clinical studies and one prospective cohort.

Two reviewers independently assessed the titles and abstracts, followed by an assessment of eligibility based on the full text. If relevant data were missing, the original authors were contacted. Disagreements between the two reviewers were resolved by discussion. If this failed, a third reviewer acted as an arbiter.

Data Extraction and Quality Assessment

Using a standardized data extraction form, two reviewers independently extracted the data. The data that was retrieved covered a wide range of topics, such as research characteristics (author, year, and design), participant demographics, intervention specifics (dosage, frequency, and duration), and outcomes evaluated (clinical remission, endoscopic improvement, and adverse events).

Reporting and Bias

This systematic review adheres to the Preferred Reporting Items for Systematic Reviews and Meta-Analyses (PRISMA) guidelines to ensure transparency and comprehensiveness in reporting [9]. Vigorous efforts were made to minimize potential sources of bias through a comprehensive search strategy, independent study selection, data extraction, quality assessment, and use of established tools.

In the following sections, we present the results derived from the selected studies, discuss their implications, and elucidate the potential significance of upadacitinib as a therapeutic intervention for Crohn’s disease.

Results

Study Selection

**Figure 1:**
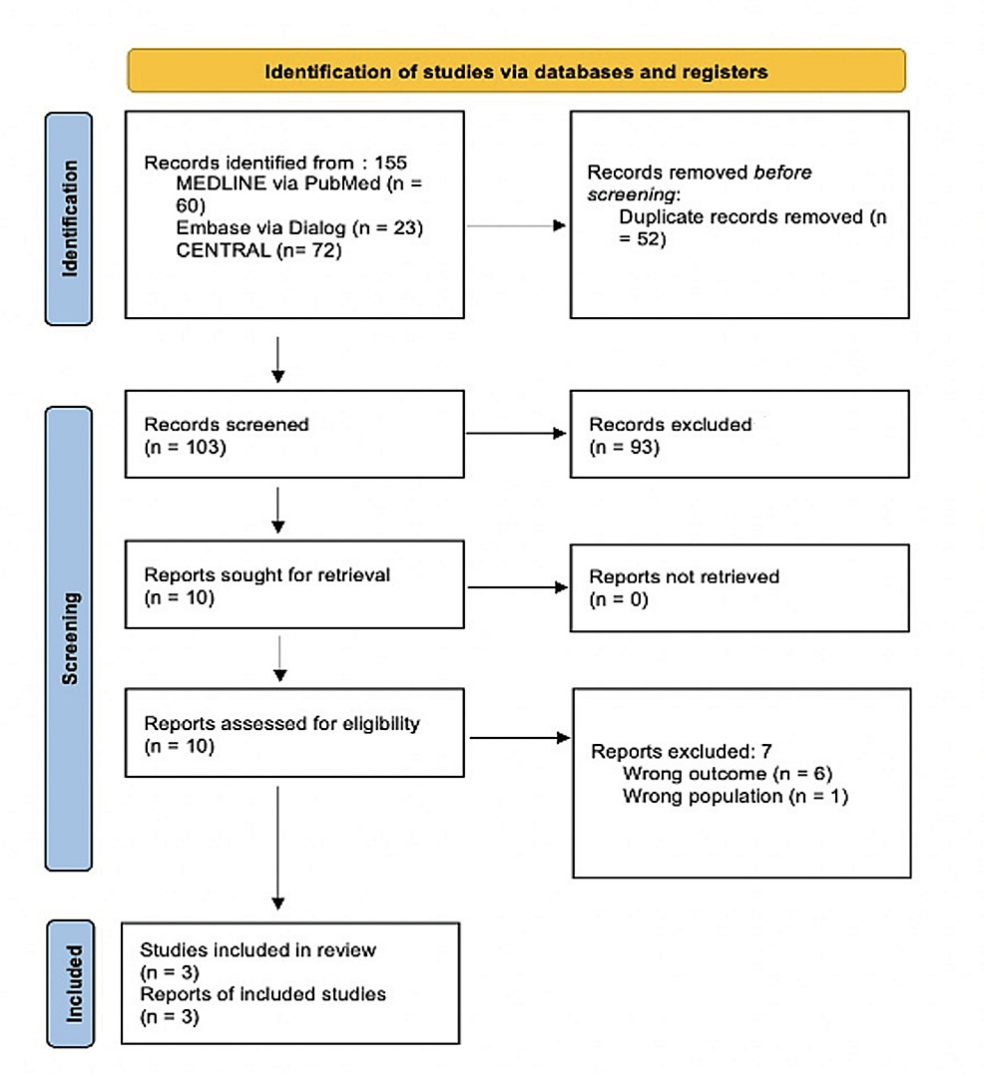
The PRISMA 2020 flow diagram. PRISMA: Preferred Reporting Items for Systematic Reviews and Meta-Analyses

A comprehensive search yielded three studies that met the inclusion criteria and were included in this systematic review (Figure 1). The selected studies encompassed a diverse range of designs, including randomized controlled trials (RCTs) and prospective cohort studies. The publication years of the included studies spanned from 2020 to 2023, reflecting the evolving landscape of upadacitinib research in Crohn's disease treatment.

Efficacy of Upadacitinib in Crohn’s Disease

The efficacy of upadacitinib in Crohn's disease management was a focal point of the selected studies. In Table 1, among the RCTs, three demonstrated statistically significant improvements in clinical remission rates in patients receiving upadacitinib compared with those in the control group [14-16]. Endoscopic outcomes were another key parameter evaluated in the selected studies, three studies reported significant improvements in endoscopic remission following upadacitinib treatment. Notably, sustained reductions in mucosal inflammation have been observed over extended follow-up periods in several studies [17-19].

**Table 1:** Study characteristics and efficacy outcomes. RCT: randomized controlled trials; CMH: Cochran-Mantel-Haenszel; CD: Crohn's disease; CIs: confidence intervals; SEC-CD: simple endoscopic score for Crohn’s disease; OLE: open-label extension; SF score: stool frequency score; AP score: abdominal pain score

First author	Year	Study design	Sample size	Dosage and frequency of upadacitinib	Duration	Primary outcome	Outcome measure	Efficacy outcome
Sandborn WJ [14]	2020	RCT	220	3 mg BID, 6 mg BID, 12 mg BID, 24 mg BID, 24 mg QID	52 weeks	16th week of clinical remission, 12th or 16th week of endoscopic remission	Multiple comparison process and modeling, as well as the Cochran-Mantel-Haenszel test, with a 2-sided threshold of 10%	Adjusted risk differences for clinical remission with Cochran-Mantel-Haenszel (CMH) corrections and their corresponding 95% confidence intervals (CIs) were as follows: 3 mg twice-daily arm 2.5 (95% CI: -12.3 to 17.3); 6 mg twice-daily arm: 16.2 (95% CI: -2.0 to 34.3); 12-mg twice-daily arm: 0.5 (95% CI: -14.1 to 15.0); 24 mg twice-daily arm: 11.2 (95% CI: -6.1 to 28.5); 24 mg once-daily arm: 4.1 (95% CI: -11.5 to 19.6). For endoscopic remission, CMH-adjusted risk differences with their corresponding 95% CIs were as follows: 3 mg twice-daily arm: 9.9 (95% CI: -0.3 to 20.1); 6 mg twice-daily arm: 7.4 (95% CI: -1.6 to 16.4); 12 mg twice-daily arm: 7.7 (95% CI: -1.5 to 16.8); 24 mg twice-daily arm; 21.0 (95% CI: 6.8 to 35.2); 24 mg once daily arm: 13.6 (95% CI: 1.8 to 25.5)
D’Haens G [15]	2022	RCT	107	15 mg QD, 30 mg QD, extended dose 30 mg QD	30 weeks	Clinical remission was consistently maintained at a rate of 2.8 per 1.0 across all groups from week 0 to month 30. Similarly, endoscopic response remained stable in all groups, with rates of 68%, 67%, and 40%, respectively, at month 24	Clinical remission, defined as an SF score of less than or equal to 2.8 and an AP score of less than or equal to 1.0, and endoscopic remission, defined as an SES-CD score of 4 or lower with a reduction of more than 2 points from the CELEST study baseline, and no subscore exceeding 1 were the criteria used for assessment	At the outset of the CELEST OLE study (week 0), clinical remission rates of 2.8/1.0 were attained by 61% of patients in the upadacitinib 15 mg group, 57% in the 30 mg group, and 45% in the upadacitinib dose-escalated group. In the upadacitinib 15 mg and 30 mg groups, these rates were sustained up to month 30 for 61% and 54% of patients, respectively. Within the dose-escalated group, 55% of patients achieved clinical remission at month 30 (as shown in Figure 1), with a median time of 254 days (range: 31-824 days) until dose escalation. The proportion of patients achieving endoscopic remission (ranging from 44% to 55%) exhibited an increase during the initial 12 months of the CELEST OLE study across all three groups based on observed case analysis. This achievement was maintained at 24 months for patients receiving upadacitinib 15 mg and 30 mg once daily, whereas it declined among patients in the dose-escalated group
Friedberg S [16]	2023	Prospective cohort	40	82% 45 mg/dL; 17.5% 15 mg/dL	8 weeks	Clinical remission at weeks 2, 4, or 8	Simple Clinical Colitis Activity Index and the Harvey-Bradshaw Index, as well as C-reactive protein and fecal calprotectin	Out of the 11 patients without any small intestine involvement in their Crohn's disease (CD), 80% of those who initially had active disease achieved both clinical response and remission by week 8. Likewise, among those with CD affecting the small intestine (L1 or L3), 75% and 66.7% of patients, respectively, achieved clinical response and remission

Safety Profile of Upadacitinib

The safety profile of upadacitinib garnered significant attention in the selected studies (Table 2). Adverse events were consistently reported, with herpes zoster, intestinal perforations, non-melanoma skin cancer, adjudicated cardiovascular events, and anemia being the most frequently observed. Instances of serious adverse events necessitating treatment discontinuation were reported in three studies, underscoring the importance of thorough safety monitoring in patients receiving upadacitinib.

**Table 2:** All the adverse events reported in each study. RCT: randomized controlled trials

Study	Year	Type of study	Outcome
Sandborn et al. [14]	2020	RCT	Herpes zoster, opportunistic infection, intestinal perforations, malignancy, excluding non-melanoma skin cancer, non-melanoma skin cancer, adjudicated cardiovascular events
D’Haens et al. [15]	2022	RCT	Herpes zoster, intestinal perforations, non-melanoma skin cancer, adjudicated cardiovascular events, anemia, neutropenia, creatine phosphokinase elevation
Friedberg et al. [16]	2023	Prospective cohort	Anemia

In summary, the synthesized evidence indicates that upadacitinib holds promise as a potential therapeutic intervention for Crohn's disease. The observed improvements in the clinical remission rates and endoscopic outcomes suggest their potential to modulate inflammation and promote mucosal healing. However, the safety profile underscores the need for careful monitoring, especially given reported adverse events. The elucidated mechanisms suggest that the effects of upadacitinib extend beyond symptom relief, potentially involving immune pathway modulation. The subsequent "Discussion section" contextualizes these findings, addresses their implications, and discusses the limitations of this systematic review.

Discussion

Synthesis of Findings

The findings of this systematic review collectively emphasize the potential role of upadacitinib as a novel therapeutic approach for Crohn's disease. Multiple trials have demonstrated substantial improvements in clinical remission rates and endoscopic results, indicating the beneficial effects of this treatment in addressing underlying inflammatory processes and supporting mucosal repair, which are particularly promising for patients with refractory Crohn's disease who do not respond adequately to conventional treatment [20-23].

Implications for Clinical Practice

The implications of upadacitinib efficacy are far-reaching. It offers a potential alternative for individuals for whom existing treatment strategies have shown limited effectiveness [24]. However, documented adverse events underscore the importance of safety considerations, emphasizing the need for careful patient selection and ongoing monitoring throughout treatment [25]. Balancing the potential benefits of the risks of serious adverse events is essential for responsible clinical decision-making.

Mechanisms of Action and Multifaceted Effects

The mechanistic insights provided by the selected studies are pivotal for understanding the multifaceted effects of upadacitinib, and the selective inhibition of JAK pathways presents a highly focused and promising avenue for effectively mitigating the intricate cascade of pro-inflammatory cytokines and immune responses. This approach has the potential to significantly dampen and regulate the complex series of events that actively contribute to the establishment and perpetuation of the chronic inflammatory characteristics of Crohn's disease, which is in strong agreement with the documented and notable enhancements observed in clinical outcomes. These compelling correlations hint at a plausible and intricate connection existing between the intricate mechanisms of upadacitinib and the highly sought-after therapeutic effects it imparts [26].

Safety Considerations

While the efficacy of upadacitinib is promising, it is important to consider its safety profile, as highlighted by the reported adverse events. This calls for a balanced approach, in which the potential benefits are weighed against safety concerns. The occurrence of infections and other adverse events highlights the crucial need for comprehensive monitoring and patient education regarding the potential risks. This emphasizes the importance of diligently tracking patients' well-being and offering them clear insights into potential drawbacks. Clinicians must weigh the benefits of these risks, particularly when considering long-term treatment strategies.

Limitations

This systematic review is not without limitations and may vary in terms of the populations studied, interventions, outcome measures, and study designs. This heterogeneity makes it challenging to combine the results and draw meaningful conclusions. The potential for publication bias and selective reporting cannot be overlooked, potentially affecting the overall interpretation of the results as there is a tendency for studies with positive or significant results to be published more often than those with negative or non-significant results. Additionally, the lack of long-term follow-up in some studies and the inclusion of studies with varying methodological qualities introduce uncertainties that impact the robustness of the conclusions.

Future Research Directions

The findings of this review will serve as a springboard for future research. Well-designed, long-term randomized controlled trials with standardized outcome measures are needed to establish the sustained efficacy and safety of upadacitinib in the context of Crohn's. Further exploration of patient subgroups and factors influencing treatment response can enhance our understanding of their applicability in clinical practice and enhance the efficacy of combination therapies with other medications, such as immunomodulators or biologics, to optimize treatment outcomes. Mechanistic studies are warranted to elucidate the precise pathways influenced by upadacitinib and can potentially inform the development of targeted therapies.

Clinical Guidelines and Patient-Centric Care

The evidence synthesized in this review contributes to the evolving landscape of Crohn's disease treatment guidelines. Randomized clinical trials have shown clinical remission in moderate-to-severe cases of upadacitinib. Evidence suggests that the drug is effective in producing endoscopic remission rather than clinical remission in the initial stages [18,19]. However, it is imperative to emphasize the individuality of each patient's condition and the need for personalized treatment decisions. While the potential of upadacitinib is promising, the patient's medical history, preferences, and potential comorbidities should be considered in patients with refractory disease who have failed to respond to at least one of the following: corticosteroids, aminosalicylates, immunosuppressants, or previous biologic therapy (infliximab, adalimumab, golimumab, vedolizumab, or ustekinumab) to ensure optimal outcomes and prevent adverse reactions.

## Conclusions

In conclusion, this systematic review provides valuable insights into the potential of upadacitinib as a therapeutic intervention for Crohn's disease. The evidence synthesized from a diverse range of studies suggests that upadacitinib holds promise in improving clinical remission rates and endoscopic outcomes, addressing the underlying inflammatory processes, and supporting mucosal healing. This is particularly encouraging for patients who have not responded adequately to conventional treatments. However, the safety profile of upadacitinib is a matter of concern, with reported adverse events, such as herpes zoster, intestinal perforations, and non-melanoma skin cancer. These findings underscore the importance of careful patient selection, ongoing monitoring, and patient education regarding potential risks associated with upadacitinib therapy. The mechanisms of action of upadacitinib, particularly its selective inhibition of JAK pathways, offer a targeted approach to modulating the complex cascade of pro-inflammatory cytokines and immune responses implicated in Crohn's disease. This mechanistic insight aligns well with the observed clinical improvements and suggests a promising avenue for future research and therapeutic development.

While this systematic review sheds light on the potential of upadacitinib, it is not without limitations, including heterogeneity among studies, potential publication bias, and varying methodological qualities. Future research should focus on well-designed, long-term randomized controlled trials with standardized outcome measures to establish the sustained efficacy and safety of upadacitinib. In clinical practice, upadacitinib may offer an alternative for individuals with refractory Crohn's disease who have not responded to conventional treatments. However, individualized treatment decisions, considering patient history, preferences, and comorbidities, are essential to optimize outcomes and minimize potential adverse reactions. Overall, upadacitinib represents a promising addition to the evolving landscape of Crohn's disease treatment, offering hope for improved management and quality of life for patients with this challenging condition. Further research and clinical experience will continue to shape its role in personalized treatment strategies for Crohn's disease.
